# Autophagy Regulation by Crosstalk between miRNAs and Ubiquitination System

**DOI:** 10.3390/ijms222111912

**Published:** 2021-11-03

**Authors:** Junyan Qu, Zhenghong Lin

**Affiliations:** School of Life Sciences, Chongqing University, Chongqing 401331, China; qujunyan1229@yeah.net

**Keywords:** microRNAs, E3 ubiquitin ligases, deubiquitinases, autophagy

## Abstract

MicroRNAs (miRNAs) are non-coding single-stranded RNA molecules encoded by endogenous genes with ~22 nucleotides which are involved in the regulation of post-transcriptional gene expression. Ubiquitination and deubiquitination are common post-translational modifications in eukaryotic cells and important pathways in regulating protein degradation and signal transduction, in which E3 ubiquitin ligases and deubiquitinases (DUBs) play a decisive role. MiRNA and ubiquitination are involved in the regulation of most biological processes, including autophagy. Furthermore, in recent years, the direct interaction between miRNA and E3 ubiquitin ligases or deubiquitinases has attracted much attention, and the cross-talk between miRNA and ubiquitination system has been proved to play key regulatory roles in a variety of diseases. In this review, we summarized the advances in autophagy regulation by crosstalk between miRNA and E3 ubiquitin ligases or deubiquitinases.

## 1. Introduction

A living cell is a complex dynamic system which can respond and adapt to environmental changes and stress all of the time. Among living cells, proteins play a key role in all physiological and pathological cellular functions. Therefore, it is of great significance to understand the synthesis, degradation, modification, and related regulation of proteins.

Ubiquitin proteasome system (UPS) is one of the main pathways regulating protein degradation in eukaryotic cells and is also a key regulatory mechanism in a variety of biological processes [1]. Ubiquitination is a reversible post-translational modification that occurs under the continuous action of E1 ubiquitin activating enzyme, E2 ubiquitin coupling enzyme, and E3 ubiquitin ligase [2,3]. Proteins labeled with ubiquitin are broken down by proteasome into smaller peptides, amino acids, and ubiquitin that can be reused [4]. In addition, ubiquitination can also serve as a marker to activate certain signals, such as autophagy and immune response [5].

MicroRNA (miRNA) is an evolutionarily conserved small non-coding RNA that is involved in the regulation of gene expression during the translation phase and is considered to be abnormally expressed in a variety of human diseases [6,7,8,9]. MiRNA can inhibit the expression of target genes at the translation level or directly lead to the degradation of mRNA through complementary binding with target mRNA [10,11,12].

Both ubiquitination and miRNAs are key regulators of protein and related signaling involved in most biological processes such as cell cycle differentiation and apoptosis, and autophagy is no exception given its central role in cellular stress and survival responses. Genes and proteins involved in autophagy pathways are also controlled by a variety of miRNAs and UPS. Moreover, autophagy related regulatory abnormalities are associated with various kinds of diseases, including cancer, neurodegeneration, and cardiovascular disease. In addition, there is crosstalk between ubiquitin ligases, deubiquitinases, and miRNAs. In this review, we will briefly describe the mutual regulation between miRNA and UPS and focus on how their crosstalk affects autophagy and forms cellular outcomes.

## 2. Overview of miRNA

In the eukaryotic genome, only a small number of genes encode proteins, and about 97% of the transcribed products are non-coding RNAs [13]. MicroRNAs (miRNAs) are ~22 nt small noncoding RNAs that are known to play an important role in the post-transcriptional regulation of messenger RNA (mRNA) [14,15]. It is estimated that more than 60% of human genes are regulated by miRNAs [16]. At the same time, studies have shown that the sequence and structure of miRNAs are highly evolutionarily conserved among different species, suggesting that miRNAs have a critical regulatory function [11,17,18].

MiRNAs are usually transcribed in the nucleus by RNA polymerase II (polII), and the initial product is a large primary miRNAs (pri-miRNA) with a 5′ 7-methyl guanosine cap and a 3′ poly adenosine tail [14]. Pri-miRNA was originally processed by Drosha in the nucleus to form a precursor miRNA (pre-miRNA) of ~70 nt that forms a hairpin, which was exported to the cytoplasm via nuclear transport receptor exportin-5 and the cofactor Ran-GTP [19]. It is then cleaved by the RNase III enzyme Dicer into a double stranded RNA of ~22 nt [20]. Under the action of the Argonaute (AGO) proteins, one strand of this duplex is selected as a mature miRNA and is then incorporated into the miRNA-induced silencing complex (miRISC) [21,22].

**Figure 1:**
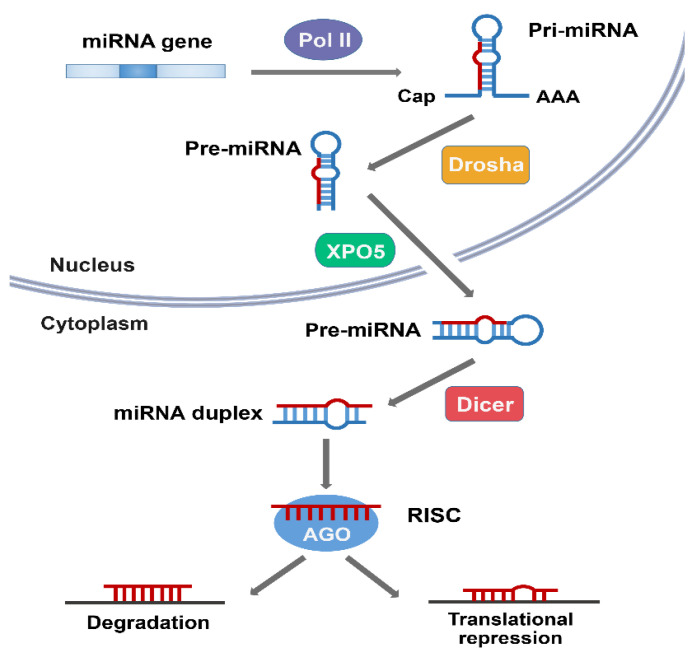
The biogenesis of microRNA (miRNA). MiRNA is transcribed by RNA polymerase II (pol II) in the nucleus as a pre-miRNA, processed by Drosha to a pre-miRNA. Pre-miRNA is then exported from the nucleus to the cytoplasm by exportin 5 (XPO5). In the cytoplasm, Dicer cleaves pre-miRNA to produce the miRNA duplex, and one strand of the resulting duplex is loaded onto the Argonaute (AGO) protein to form a miRNA-induced silencing complex (miRISC), which targets mRNAs for regulation. MiRNAs that form perfect duplexes with their targets direct degradation and those that support partial duplexes inhibit protein expression.

MiRISC directs the miRNA to binding sites in the target mRNAs, which usually leads to gene repression [22]. If the miRNA is completely complementary to the target site, the binding of these miRNAs often leads to degradation of the target mRNA. MiRNAs that are not completely complementary to the target mRNA usually inhibit the expression of the target gene at the protein translation level without affecting the mRNA stability (Figure 1) [10,23].

A miRNA can have multiple target genes, and several miRNAs can jointly regulate the same gene [15,23]. Therefore, the wide variety of biological functions of miRNAs is not surprising. Although the important roles of miRNAs have been demonstrated in several studies, the research on miRNAs is still in its infancy and only a small part of their biological functions has been elucidated [9,24,25,26,27].

## 3. Overview of Ubiquitin-Proteasome System (UPS)

Ubiquitin (Ub) is a 76-amino-acid protein highly conserved among all eukaryotes. It covalently binds to the lysine residues of the substrate protein and acts as a signal molecule to mediate its degradation or regulate its biological functions [2]. Ubiquitin contains seven lysine residues (K6, K11, K27, K29, K33, K48, and K63) and one N-terminal methionine residue, each of which can be attached to another ubiquitin moiety [3]. As a consequence, the modification of ubiquitin can form ubiquitin chains of different lengths and linkage types. The lysine binding sites of ubiquitin determine different cellular functions and protein fates, including degradation, signal transduction, and altered subcellular localization [5]. Among them, K48- and K11-linked chains mediate the recognition and degradation of ubiquitinated substrate proteins by 26S proteasome, whereas other linked sites, such as K63, do not lead to degradation but regulate other cellular processes, such as DNA repair, mitochondrial genetics or NF-κB signaling pathways [28]. The physiological functions of other atypical ubiquitination modifications (K6, K27, K29, and K33) are unknown but have been of interest to researchers.

**Figure 2:**
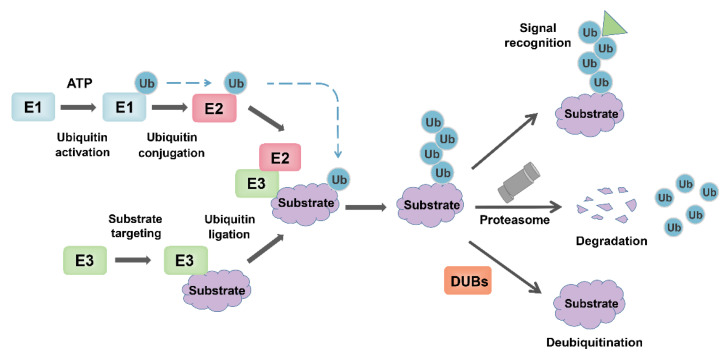
The overview of the ubiquitin-proteasome system. Ubiquitin is activated and bound to E1 in an ATP-dependent manner. Then, the activated ubiquitin is transferred to the E2, while the substrate protein to be degraded is specifically targeted by E3 ubiquitin ligase, and ubiquitin is ligated to the substrate. Ubiquitinated substrate proteins are recognized by the 26S proteasome and degraded into small peptides and amino acids. In contrast, DUB reverses ubiquitination by removing the polyubiquitin chains of proteins and maintains intracellular ubiquitin levels. In addition, some ubiquitination modifications that do not lead to degradation induce related biological effects through signal recognition, such as kinase activation, localization changes.

Ubiquitin binding is a multistep reaction that requires the sequential action of three enzymes (E1 Ub-activating enzyme, the E2 Ub-conjugating enzyme, and the E3 ubiquitin ligase) [1]. In the presence of ATP, E1 activates Ub and transfers it to E2, whose active site cysteine forms a thioester bond with the C-terminal carboxyl group of Ub. The E3 enzyme mediates the last step of Ub transfer through simultaneous interaction with a Ub-loaded E2 enzyme and a specific substrate, and finally forms the ubiquitinated substrate (Figure 2) [4,29]. Given the substrate recognition and substrate specificity of E3 ligase, its role in ubiquitin modification is particularly critical. There are more than 600 known E3 ligases in human, and they can be grouped into three categories according to their conserved domains: HECT E3 (homologous to the E6-associated protein carboxyl terminus) [30], RING finger E3s [31], and RBR (RING between RING) type E3s [32].

Ubiquitination is a reversible process that can be reversed by a specific group of enzymes called deubiquitinases (DUBs) [33]. There are about 100 DUBs encoded by the human genome, which are mainly divided into six classes: ubiquitin-specific Proteases (USPs); ubiquitin carboxy-terminal hydrolases (UCHs); ovarian-tumor proteases (OTUs); Machado-joseph disease Protein Domain Proteases (MJDs), JAMM/MPN domain-associated metallopeptidases and monocyte chemotactic protein-induced protein (MCPIP). The most abundant sub-family of DUBs is the USPs with over 50 members [34,35].

## 4. Overview of Autophagy System

Autophagy is a stress-responsive catabolic process that degrades intracellular components through lysosomal enzymes [36]. In normal physiological state, only a small amount of autophagy occurs in cells to maintain homeostasis. When cells are stimulated by intracellular and extracellular factors such as starvation, hypoxia, pathogen invasion, etc. [37], a large number of autophagy can be induced through the transduction of cell signaling pathways [38]. Thus, autophagy is a pivotal actor in development, immune response, as well as metabolic regulation and has been shown to be associated with cellular modifications related to senescence, with most studies now suggesting that a reduced autophagic potential is one of the factors of cell senescence [36,39,40]. Autophagy not only removes protein aggregates, but also damages organelles and plays a role in quality control of the cytoplasm such as mitophagy [41]. In the case of damaged mitochondria, mitophagy removes malfunctioning mitochondria to maintain the population at an optimal state. In recent years, mitophagy has received increasing attention since mitochondrial dysfunction is at the foundation of numerous diseases and a growing number of studies also suggested mitophagy as a therapeutic target [42,43].

Autophagy is composed of several closely related steps including autophagy initiation, autophagosome maturation, and autophagolysosome fusion, which involves many important autophagy-related proteins and complexes [44]. These core autophagy proteins include the following parts: (1) the ATG1/ULK1 complex, including ATG1, ATG11, ATG13, ATG17, ATG29, and ATG31, is the only core protein complex with serine/threonine kinase activity in autophagy signaling pathway. The ULK1 complex acts as a bridge connecting upstream energy receptors mTOR and AMPK with downstream autophagosomes in vivo [45,46], and plays an important role in autophagy initiation [47,48]; (2) the PI3K complex, including Vps34, Vps15, ATG6/Beclin1, and ATG14, catalyze the conversion of the lipid molecule PI to PI3P, thereby recruiting the protein that binds to PI3P [49]. Vps34 is the class III PI3K in mammals. Vps34 is activated by binding to Vps15 and further binds to Beclin1 to form the Vps34-Vps15-Beclin1 complex. During autophagy, Vps34-Vps15-Beclin1 binds to a variety of autophagy-related proteins. For example, ATG14 is combined with to Vps34-Vps15-Beclin1 to form ATG14-Vps34-Vps15-Beclin1, which is involved in the formation of autophagic vesicles [50]; the (3) ATG9 and WIPI/ATG2-ATG18 complex. ATG9 is a transmembrane protein with six transmembrane domains, which may play a role in regulating autophagy by affecting vesicular transport [51]. ATG9 circulates in autophagic vesicles and cytoplasm, depending on ATG17 or ATG11 complex to locate PAS, and ATG2-ATG18 complex to leave PAS [52,53]. In mammals, specific silence of mATG9 gene can inhibit the formation of autophagic vesicles and protein degradation, and inhibit the occurrence of autophagy; and (4) ubiquitin-like systems ATG12-ATG5 and ATG8/LC3. There are two ubiquitin-like binding pathways involved in autophagosome formation. Both ATG8 and ATG12 are ubiquitin-like proteins, ATG12 can covalently bind with ATG5, and ATG8 can covalently bind with the lipid molecule phosphatidylethanolamine PE [54,55]. Similar to the ubiquitin system, ATG12 is transmitted by ATG7 to ATG10, which eventually binds to the lysine side chain of ATG5 and forms a complex with ATG16, promoting the exposure of membrane-binding sites on ATG5. Similarly, ATG7 transfers ATG8 to ATG3 [56]. With the help of ATG12-ATG5-ATG16, LC3 conjugates to lipid molecule phosphatidylethanolamine (PE), promoting isolation membrane expansion and autophagic vesicle completion [57]. These key proteins in the complex autophagy regulatory network are regulated by a variety of molecular signals, including ubiquitin ligases, deubiquitinases and miRNAs [58].

## 5. MiRNAs Are Involved in Autophagy via Regulation of E3 Ubiquitin Ligases

E3 ubiquitin ligase specifically recognizes substrates and induces substrate protein degradation, and most of the key proteins involved in autophagy are regulated by ubiquitin ligase. Therefore, it is of great significance to understand the ubiquitination mechanism in autophagy. At the same time, miRNA often acts as an upstream regulator of E3 ubiquitin ligase, co-regulating autophagy with ubiquitin ligase (Table 1).

### 5.1. MARCH5 Regulates Autophagy in a Mir-30a-Dependent Manner

The membrane-associated RING-CH (MARCH) protein belongs to the RING Finger protein family of E3 ubiquitin ligases and consists of 11 members [66]. Among them, MARCH5 is an integral mitochondrial outer membrane protein with four transmembrane segments [67]. MARCH5 participates in the control of mitochondrial morphology and plays a key role in the growth of cells and tissues [68,69]. In addition, studies have shown that MARCH5 can promote mitophagy through its ubiquitination [70,71]. In addition, MARCH5 also participates in the regulation of apoptosis [72,73] and maintains the stemness of mouse embryonic stem cells [74,75].

**Figure 3:**
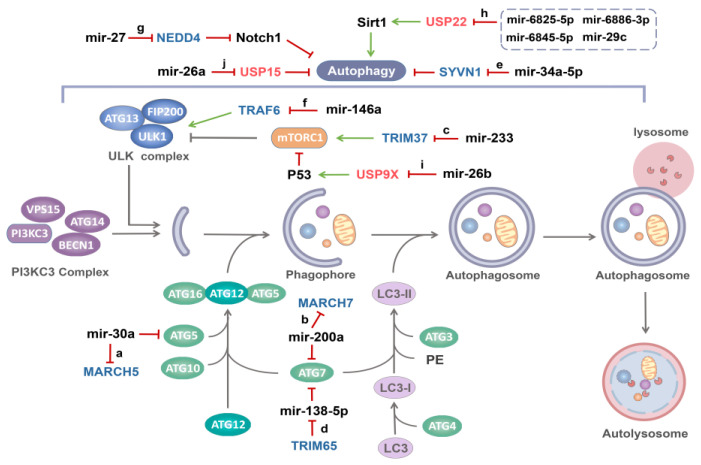
MiRNAs regulates autophagy by targeting E3 ubiquitin ligases or DUBs. Once receiving autophagy induction signals, ULK1 complex mediates the initiation of autophagy, and PI3KC3 complex participates in the nucleation of phagophore. Under the action of two ubiquitin-like systems, phagophore is continuously extended to form autophagosome, which eventually fused with lysosome to form autolysosome and the contents of the autolysosome are then degraded and exported back into the cytoplasm for reuse by the cell. (a) Mir-30a targets both MARCH5 and ATG5, MARCH5 RNA serves as a competing endogenous RNA (ceRNA) to regulate the expression of ATG5 by competing with ATG5 for mir-30a. (b) ATG7 and MARCH7 mRNA, as ceRNAs, regulate each other by competing for mir-200a. (c) Mir-233 increases autophagy by negatively targeting TRIM37, which promotes the mTORC1 pathway. (d) As a ubiquitin ligase of TNRC6, a component of miRISC, TRIM65 blocks the function of miRISC to achieve the effect of inhibiting mir-138-5p. However, ATG7 was upregulated by TRIM65 as a target of mir-138-5p. (e) Mir-34a-5p promotes autophagy by directly targeting SYVN1. (f) Mir-146a inhibits E3 ubiquitin ligase TRAF6, which has a positive regulatory effect on ULK1 protein through K63-linked ubiquitination. (g) Mir-27 targets NEDD4, the E3 ubiquitin ligase of Notch1, increasing Notch1 protein expression and decreasing autophagy. (h) Mir-6825-5p, mir-6845-5p, mir-6886-3p and mir-29c can directly target USP22, the DUB of Sirt1, and ultimately suppress Sirt1-mediated autophagy. (i) Mir-26b negatively targets USP9X, a DUB of p53, and affects autophagy by inducing p53 degradation. (j) Mir-26a negatively targets USP15, which inhibits autophagy.

Recent studies have shown that the expression of MARCH5 in epithelial ovarian cancer tissue is higher than that in normal ovarian tissue, and the up-regulated MARCH5 promotes autophagy in epithelial ovarian cancer cells, which is beneficial to cancer progression. Interestingly, MARCH5 regulates autophagy in a mir-30a-dependent manner. MARCH5 has been shown to be the target gene of mir-30a, and ATG5 and SMAD2, which are involved in autophagy signaling, have also been identified as target genes of mir-30a [59,76,77]. At this point, MARCH5 mRNA can serve as a competing endogenous RNA (ceRNA) to regulate the expression of SMAD2 and ATG5 by competing mir-30a, and once mir-30a is inhibited, the regulatory effects of MARCH5 on ATG5 and SMAD2 will also be eliminated (Figure 3a) [59]. Therefore, MARCH5 serves as the target of mir-30a, and together with mir-30a regulates the autophagy through ATG5 and SMAD2.

### 5.2. MARCH7 and Mir-200a Jointly Regulate Autophagy

The E3 ubiquitin ligase MARCH7, a member of the MARCH family, plays an important role in T cell proliferation and neuronal development [78,79]. MARCH7 is highly expressed in ovarian cancer cells and promotes ovarian cancer cell proliferation [80].

Recent studies have found that the expression level of ATG7 in ovarian cancer tissues is well correlated with MARCH7. Mir-200a is believed to be the miRNA shared by MARCH7 and ATG7, and their direct binding to mir-200a was also confirmed by luciferase assay. ATG7 expression was more pronounced in tumor tissue than in normal ovarian tissue. On one hand, MARCH7 silencing down-regulated ATG7 expression. However, this regulation was abolished when mir-200a was silenced [60]. MARCH7 mRNA may function as a competing endogenous RNA (ceRNA) to regulate the expression of ATG7 by competing with mir-200a (Figure 3b). On the other hand, MARCH7 overexpression promoted TGF-β induced autophagy through regulating TGF-β-smad2/3 pathway [60,81,82]. Therefore, mir-200a inhibits TGF-β induced autophagy of SKOV3 cells by targeting MARCH7.

### 5.3. Mir-233 Induces Autophagy by Inhibiting TRIM37

TRIpartite Motif (TRIM) proteins are part of the largest subfamily of E3 ligases, consisting of a RING domain, B-box motif, and a core-coil region [83]. TRIM37 is one of the TRIM members and is characterized by the presence of a unique MATH domain in the C-terminal portion. TRIM37 is often overexpressed in a variety of cancer cells [84,85,86], and it has been confirmed that overexpression of TRIM37 induces cell proliferation [87]. Perplexingly, however, mutations in the TRIM37 gene cause Mulibrey nanism, an inherited growth disorder [88,89]. This may indicate that the expression of TRIM37 in normal cells requires an optimal balance.

Current studies show that the regulation of TRIM37 on disease may be related to autophagy, for example, TRIM37 depletion increases the basal autophagy flux through the MTORC1 pathway [90]. Consistent with this, TRIM37 knockdown can increase the expression of LC3II and promote autophagy in chondrocytes, while overexpression of TRIM37 can inhibit autophagy [61]. In addition, recent studies have found that mir-223, a regulatory factor upstream of TRIM37, specifically binds to 3′-UTR binding site of the TRIM37 mRNA in a complementary way to regulate its expression (Figure 3c) [61].

Under the overexpression condition (mimic mir-223), the expression of TRIM37 decreased by approximately one-third [61], indicating that other factors are probably involved in the regulation of TRIM37, since it is well known that the UTR of mammalian mRNA is usually targeted by more than one miRNA. In conclusion, autophagy plays a critical role in the development and viability of chondrocytes during endochondral ossification, and mir-223 can inhibit the key gene TRIM37, thereby regulating autophagy and cell proliferation. However, how TRIM37 affects the autophagy pathway needs further study. The crosstalk between mir-233 and TRIM37 may provide us a clue for the treatment of related disease.

### 5.4. TRIM65 Affects Autophagy through Mir-138-5p/ATG7

Autophagy has been identified as one of the key mechanisms of chemotherapeutic resistance, and regulation of autophagy has become a promising strategy to overcome chemotherapeutic resistance in cancer therapy [91,92]. TRIM proteins are involved in the regulation of carcinogenesis, autophagy and chemoresistance [93], for instance, TRIM32 in breast cancer [94], and TRIM14 in gliomas [95]. TRIM65 is often overexpressed in cancer tissues and is considered to be an oncogenic protein [96,97,98,99]. It has been reported that TRIM65 is an E3 ubiquitin ligase for trinucleotide repeat containing six (TNRC6) proteins, which is a component of RNA-induced silencing complex (RISC), and participates in miRNA-induced gene silencing [100]. Therefore, TRIM65 can relieve miRNA-driven suppression of mRNA expression through ubiquitination and subsequent degradation of TNRC6 (Figure 3d).

Recent studies have shown that TRIM65 is involved in the regulation of autophagy and chemical resistance. Knockdown of TRIM65 can significantly down-regulate the expression of ATG7, an important autophagy mediator, to inhibit autophagy in A549/DDP cells. Meanwhile, the expression of mir-138-5p in NSCLC tissues was negatively correlated with TRIM65 mRNA, and mir-138-5p was significantly increased after TRIM65 knockdown [65]. Importantly, ATG7 was identified as a target gene of mir-138-5p, which affects autophagy by down-regulating ATG7 expression [101]. Moreover, mir-138-5p inhibitor significantly eliminated the effects of TRIM65 knockdown on cisplatin-induced autophagy and apoptosis, which further proved that the down-regulation of TRIM65 on ATG7 was mediated by mir-138-5p [65]. In other words, TRIM65 induces ubiquitination and degradation of TNRC6A, leading to inhibition of mir-138-5p expression, thus affecting ATG7 and autophagy and TRIM65 may be a potential therapeutic target due to its new functions of miRNA-mediated autophagy and cisplatin resistance.

### 5.5. Mir-34a-5p Targets SYVN1 to Induce Autophagy

Mir-34a-5p has been reported to be closely associated with the occurrence and development of many diseases [102,103,104], including the regulation of autophagy. For example, mir-34a-5p overexpression activates Beclin1 by inhibiting Bcl-2 and participates in autophagy induction [105]. Recent study has identified SYVN1 as a target of mir-34a-5p, which directly targets SYVN1 in 293T cells and inhibits the expression of SYVN1 mRNA and protein (Figure 3e) [62].

SYVN1 expression was decreased in osteoarthritis tissues. Transfection of anti-mir-34a-5p significantly induced SYVN1 expression, inhibited cell apoptosis and autophagy, and promoted cell proliferation. Meanwhile, SYVN1 knockdown impaired the effect on cell proliferation, apoptosis and autophagy caused by anti-mir-34a-5p [62]. SYVN1 is involved in endoplasmic reticulum stress, chronic inflammation, immunity, tumor regulation and other aspects [106,107,108,109,110]. Currently, the specific mode of SYVN1 regulation of autophagy is not clear, which may need further investigation in autophagy regulation.

### 5.6. Role of TRAF6 as a Target of mir-146a in Autophagy

TRAF6 (TNF receptor-associated factor 6) is a well-known ubiquitin ligase containing a ring finger domain and five zinc finger domains at the N-terminus and a TRAF domain at the C-terminus [111,112]. TRAF6 can integrate with multiple kinases to regulate signaling pathway by functioning as an E3 ubiquitin ligase, which is closely related to malignant tumors [113], immune inflammation [114] and nervous system diseases [115].

Nucleophosmin (NPM1) mutations are considered as one of the most common genetic alteration in acute myeloid leukemia (AML) [116], and aberrant cytoplasm-dislocated NPM1 mutant is a distinct biological characterization of this disease [117,118]. It has been reported that increased autophagy activity and autophagy activation of NPM1 mutants contribute to the survival of leukemia cells [119]. The core autophagic protein ULK1 plays an indispensable role in autophagosome formation [120] and is highly expressed in NPM1 mutated AML. K63-linked ubiquitination mediated by the E3 ubiquitin ligase TRAF6 is a crucial posttranslational modification for ULK1 protein. Unlike K48 linkage, K63-linked ubiquitination has a positive effect on protein stability of ULK1 and its participation in autophagosome formation [63].

Tang et al. found that NPM1 mutations promote TRAF6-dependent K63 ubiquitination through mir-146a and further maintain the stability and kinase activity of ULK1 (Figure 3f). NPM1 mutations inhibited mir-146a and upregulated TRAF6 expression, as a direct target of mir-146a, increased K63 ubiquitination of ULK. Subsequently, high expression of ULK1 mediates autophagy activation and promotes leukemia cell proliferation. In contrast, in Dengue disease, mir-146a inhibits autophagy by targeting TRAF6, leading to ubiquitination of IFN-β rather than through ubiquitination of ULK1 [121,122]. In chondrocytes, hypoxia induces up-regulation of mir-146a and inhibits its target TRAF6, possibly regulating autophagy through the NF-κB signaling pathway [123]. This suggests that there may be different modes of action in different diseases.

### 5.7. Mir-27 Targets NEDD4 to Reduce Autophagy

Mir-27 has been described as a carcinogen and is frequently overexpressed in a number of human tumors, including osteosarcoma [124], gastric cancer [125] and thyroid cancers [126]. In multiple myeloma, high expression of mir-27 was observed to predict a poor prognosis and increase cancer progression in humans [127]. Autophagy has been shown to contribute to chemotherapy sensitivity in a number of studies, but the autophagy level of multiple myeloma tumor cells is significantly lower than that of normal myeloid plasma cells [64]. Recent studies have reported the regulatory mechanism of mir-27 on autophagy in multiple myeloma.

Mir-27 negatively targets the ubiquitin ligase NEDD4, which specifically binds Notch1 to increase the ubiquitination of Notch1 in multiple myeloma cells (Figure 3g). mir-27 or Notch1 overexpression or NEDD4 silencing diminished autophagy but enhanced proliferation and invasion of multiple myeloma cells. Collectively, mir-27 elevated Notch1 expression by targeting NEDD4 and promoted the development of multiple myeloma by inhibiting cell autophagy, thus providing a scientific basis for innovative treatment of multiple myeloma [64].

## 6. MiRNAs Are Involved in Autophagy via DUBs Regulation

As part of the ubiquitin-proteasome system, the role of deubiquitinases is equally important. Deubiquitinases stabilize substrate proteins and participate in the regulation of autophagy related signaling pathways by removing ubiquitin chains of substrates. There was also an interesting crosstalk between MiRNA and deubiquitinases (Table 2).

### 6.1. MiRNAs Regulate Autophagy via USP22

Ubiquitin specific proteases (USPs) are the largest family of ubiquitination enzymes, which play an important role in the ubiquitination system by specific depolymerization of ubiquitin from ubiquitinated substrates [132]. USP22 is expressed in most normal human tissues but is overexpressed in malignancies, such as colorectal, liver, breast, stomach and lung cancers, and has been shown to be associated with tumor progression [133,134,135,136,137]. USP22 is considered to be a target for cancer therapy, and is involved in a variety of cancer regulatory pathways, such as C-Myc, p53 and WNT pathway [138,139,140]. The role of USP22 in autophagy has also attracted attention and the activation of ERK1/2 was identified to be one of the mechanisms underlying the promotion of LC3 processing by USP22 [141].

At present, several miRNAs have been shown to bind USP22 mRNA and inhibit its protein expression. In pancreatic cancer cells, mir-29c directly targets and down-regulates USP22, increasing chemotherapy sensitivity and inducing apoptosis by inhibiting USP22-mediated autophagy [128]. In hepatocellular carcinoma cells, three miRNAs (mir-6825-5p, mir-6845-5p, and mir-6886-3p) can directly target USP22, the deubiquitination enzyme of SIRT1, and ultimately affect autophagy (Figure 3h) [129]. It is well known that SIRT1 induces autophagy by regulating many key autophagy components, such as ATG5, ATG7, ATG8, Beclin1, p53 [142]. Down-regulation of mir-6825-5p, mir-6845-5p, and mir-6886-3p stabilize SIRT1 by enhancing the deubiquitination effect of USP22, and play a key role in SIRT1-mediated autophagy.

### 6.2. Mir-26b Affects Autophagy via USP9X/p53 Ubiquitination Degradation Pathway

MirRNA-26 is involved in the occurrence and progression of a variety of diseases [143,144,145], among which mir-26b has been shown to be a tumor suppressor in most studies and has been proved to be related to the sensitivity of cancer chemotherapy in several studies, such as improving the sensitivity of colon cancer cells to 5-FU [146], inhibiting the resistance of gastric cancer cells to paclitaxel [147], and enhancing the potency of cisplatin in lung cancer cells [148]. Chemotherapy resistance is often associated with autophagy. In fact, inhibition of autophagy has been shown to overcome chemoresistance in many tumor cells [149,150,151]. Previously, mir-26a/b has been reported to enhance apoptosis and sensitivity of HCC cells via inhibiting ULK1 [151]. Mir-26 inhibits autophagy in non-small cell lung cancer cells via inhibiting the TGF-β1-JNK signaling pathway [152].

Mir-26b is encoded at 9P21.3, a vulnerable site in the genome, which has been reported to be missing in many HCC tumors and down-regulated in HCC cells [153]. In addition, upregulation of mir-26b can slow cell proliferation and migration in HCC cells and enhance sensitivity to adriamycin by inhibiting autophagy in HCC cells [151,154]. Chen et al. showed that the expression of mir-26b was down-regulated after adriamycin treatment in human HCC cells. While mir-26b mimic inhibited autophagy and enhanced the sensitivity of HCC cells to adriamycin. Interestingly, this effect disappeared in p53 deficiency of Hep3B cells. However, mir-26b mimic did not directly affect the expression of p53, suggesting that the regulation of p53 by mir-26b requires the mediations of other molecules [130].

Previous studies have reported that WP1130 enhances the sensitivity of HCC cells to adriamycin by USP9X-dependent p53 degradation [155]. Therefore, luciferase assay confirmed that mir-26b could directly bind USP9X, which is a member of the USP family. Mir-26b targets USP9X, the deubiquitination enzyme of p53, and induces the degradation of p53, thereby affecting autophagy and HCC cell drug resistance (Figure 3i). Meanwhile, MG132, as a proteasome inhibitor, reversed the inhibition of p53 expression by mir-26b in HCC cells under the action of adriamycin, which further confirmed that the down-regulation of p53 was achieved through proteasome-dependent degradation via p53 ubiquitination [130]. Mir-26b/USP9X/p53 ubiquitination degradation pathway may represent a potential gene-targeting approach for HCC treatment in the future.

### 6.3. Mir-26a Targets USP15 to Activate Autophagy

Autophagy is known to be widely involved in the development of various diseases, and in addition to cancer, autophagy also plays an important role in cardiovascular diseases. A large number of studies have found that autophagy is regarded as a protective factor against ischemic injury in cardiomyocytes and is involved in cardiac cell death associated with acute myocardial infarction (AMI) [156,157,158].

Recent studies have shown that mir-26a is a highly conserved miRNA that is dysregulated in a number of cardiovascular diseases [159,160]. In a mouse model of myocardial infarction, inhibition of mir-26a leads to cardiac injury in vitro and in vivo, whereas mir-26a overexpression attenuated ischemic stress-induced cell death by activating autophagy. The signaling mechanism of mir-26a’s cardiac protective effect is mediated by inhibition of USP15 [131].

USP15 can inhibit autophagy, and in previous studies, USP15 inhibits PARKIN-mediated mitophagy through its deubiquitination enzyme activity [161,162]. In another study, mir-26a was found to directly target USP15 and activate autophagy, thereby alleviating ischemic stress-induced cardiac injury (Figure 3j). However, the specific mechanism of USP15 inhibited autophagy in cardiomyocytes remains unclear. Inhibition of mir-26a leads to a decline in cell viability that could be rescued by USP15 knockdown. Silencing USP15 also attenuated the inhibitory effect of mir-26a inhibition on autophagosome and autolysosome formation, suggesting that mir-26a regulates the autophagy of cardiomyocytes by targeting USP15 [131].

## 7. Discussion

The biological events that occur in cells are extremely complex and variable. MiRNAs are central regulators of biological events. And ubiquitin–proteasome systems are also involved in almost all cell signaling regulation such as cell cycle, cell proliferation, apoptosis, and autophagy [163,164,165]. Here, we illustrate the important impact of crosstalk between miRNA and UPS in autophagy regulation due to the increasingly important impact status of autophagy in malignant diseases such as tumors today. It is also highlighted how the dysregulation of autophagy jointly mediated by miRNA and UPS is associated with a number of human diseases and the potential of targeting these pathways for disease intervention.

Regarding the crosstalk between miRNAs and the UPS, the effects of miRNAs on the UPS are more common and easily understood, usually relying on the specific targeting of miRNAs to produce inhibition of E3 ubiquitin ligases or DUBs. Interestingly, the UPS has also been shown to exert an effect on miRNA expression that is usually indirectly regulated by acting on functional proteins involved in the maturation process of miRNAs. In addition, miRNA degradation mechanisms widely control miRNA levels in mammalian cells. MiRNA degradation mechanisms are still unclear, and some studies suggest that E3 ubiquitin ligases may be involved in part of the miRNA degradation mechanism [166,167]. Whether eradicating problematic miRNAs from cells or retaining beneficial miRNAs, this may represent a new gene regulation-based solution to disease.

A large number of studies have shown that autophagy is involved in the occurrence and development of most diseases, and autophagy regulation is also being considered for the treatment of diseases, especially in cancer. Autophagy abnormalities in these diseases are often associated with expression dysregulation of ubiquitin ligases, DUBs, and miRNAs. The initiation and nucleation steps of autophagosome formation are mostly regulated by ubiquitination, which means that ubiquitination controls the initiation of autophagy under various stress conditions. However, upstream regulatory mechanism of ubiquitination is still very intricate, and miRNAs can act as upstream regulatory factors of E3 ubiquitin ligases and DUBs. Moreover, crosstalk between miRNAs and ubiquitin system may provide new ideas for autophagy signal regulation network. At the same time, ubiquitination may also be a regulatory factor of miRNAs, generally through the ubiquitin modification of some key miRISC proteins. In addition, it is also feasible to study the upstream regulatory pathways of miRNA, such as circular RNA (circRNA) and long non-coding RNA (lncRNA).

MiRNAs regulate autophagy in a variety of cell types under different physiological conditions and in response to various stress stimuli by directly or indirectly modifying the expression of autophagy-related proteins and pathways. Based on the characteristics of miRNAs in previous studies, some miRNAs often have multiple targets and regulate autophagy through diversified signaling pathways, which may be due to different cell types or various stimulating signals caused by diseases. Similarly, different miRNAs have also been reported to control the same key protein. Therefore, the role of miRNA in autophagy regulation network is extremely complex. In order to apply miRNA and autophagy regulatory network to the treatment of diseases, it is necessary to conduct in-depth analysis of relevant targets and specific cellular environment. In the future, a further comprehensive understanding of how ubiquitination and miRNA mediate autophagy regulation will help to elucidate the pathology of human disease and develop less toxic and more specific drugs.

## Figures and Tables

**Table 1:** MiRNAs and E3 ligases involved in autophagy.

MiRNA/E3	Target	Function	References
Mir-30a	MARCH5	MARCH5 mRNA acts as ceRNA of ATG5	[59]
Mir-200a	MARCH7	MARCH7 mRNA acts as ceRNA of ATG7	[60]
Mir-233	TRIM37	Promotes autophagy by inhibiting MTORC1	[61]
Mir-34a-5p	SYVN1	Induces autophagy	[62]
Mir-146a	TRAF6	Inhibits autophagy via ULK1 protein	[63]
Mir-27	NEDD4	Attenuates autophagy through Notch1	[64]
TRIM65	Mir-138-5P	Upregulates ATG7 by inhibiting miRISC	[65]

**Table 2:** MiRNAs and DUBs involved in autophagy.

MiRNA/E3	Target	Function	References
Mir-29c	USP22	Inhibits autophagy	[128]
Mir-6825-5p	USP22		
Mir-6845-5p		Inhibits SIRT1-mediated autophagy	[129]
Mir-6886-3p			
Mir-26b	USP9X	Suppresses Autophagy by inhibiting p53	[130]
Mir-26a	USP15	Activates autophagy	[131]

## Data Availability

Not applicable.

## Abbreviations

DUBs	Deubiquitinases
UPS	Ubiquitin proteasome system
mRNA	Messenger RNA
Pri-miRNA	Primary miRNA
Pre-miRNA	Precursor miRNA
polII	polymerase II
XPO5	Exportin 5
AGO	Argonaute
TNRC6	Trinucleotide Repeat Containing Adaptor 6
miRISC	miRNA-induced silencing complex
3′-UTR	3′-Untranslated Region
RAN-GTP	RAs-related nuclear protein-GTP
NF-κB	Nuclear factor of kappa light polypeptide gene enhancer in B cells
ceRNA	Competing endogenous RNA
ULK1	Unc-51 like autophagy activating kinase 1
mTOR	mammalian target of rapamycin
AMPK	Adenosine 5′-monophosphate (AMP)-activated protein kinase
PI3K	Phosphatidylinositol 3-kinase
PI3P	Phosphatidylinositol 3-phosphate
WIPI	WD repeat domain, phosphoinositide interacting
LC3	Microtubule-associated protein 1 light chain 3
PAS	Preautophagosomal structure
SMAD2	SMAD family member 2
TGF-β	Transforming growth factor-β
NPM1	Nucleophosmin 1
AML	Acute Myeloid Leukemia
C-Myc	Transcriptional regulator Myc-like
JNK	C-Jun NH2-terminal kinase
